# Dexamethasone treatment did not exacerbate Seneca Valley virus infection in nursery-age pigs

**DOI:** 10.1186/s12917-018-1693-8

**Published:** 2018-11-20

**Authors:** Alexandra Buckley, Nestor Montiel, Baoqing Guo, Vikas Kulshreshtha, Albert van Geelen, Hai Hoang, Christopher Rademacher, Kyoung-Jin Yoon, Kelly Lager

**Affiliations:** 1 U.S. Department of Agriculture, Oak Ridge Institute for Science and Education and National Animal Disease Center, Ames, IA USA; 2 Present address: U.S. Department of Agriculture, Avian Viruses Section, Diagnostic Virology Laboratory, National Veterinary Services Laboratories, Animal and Plant Health Inspection Service, Ames, IA USA; 3 Department of Veterinary Diagnostic and Production Animal Medicine, College of Veterinary Medicine, Iowa State University, Ames, IA USA; 4 Present address: Toxikon Corporation, Bedford, MA USA; 5 U.S. Department of Agriculture, Virus and Prion Research Unit, National Animal Disease Center, Agricultural Research Service, 1920 Dayton Avenue, PO Box 70, Ames, IA 50010 USA

**Keywords:** Dexamethasone, Seneca Valley virus (SVV), Vesicular disease, Swine, Nursery-age pigs

## Abstract

**Background:**

*Senecavirus A*, commonly known as Seneca Valley virus (SVV), is a picornavirus that has been infrequently associated with porcine idiopathic vesicular disease (PIVD). In late 2014 there were multiple PIVD outbreaks in several states in Brazil and samples from those cases tested positive for SVV. Beginning in July of 2015, multiple cases of PIVD were reported in the United States in which a genetically similar SVV was also detected. These events suggested SVV could induce vesicular disease, which was recently demonstrated with contemporary US isolates that produced mild disease in pigs. It was hypothesized that stressful conditions may exacerbate the expression of clinical disease and the following experiment was performed. Two groups of 9-week-old pigs were given an intranasal SVV challenge with one group receiving an immunosuppressive dose of dexamethasone prior to challenge. After challenge animals were observed for the development of clinical signs and serum and swabs were collected to study viral shedding and antibody production. In addition, pigs were euthanized 2, 4, 6, 8, and 12 days post inoculation (dpi) to demonstrate tissue distribution of virus during acute infection.

**Results:**

Vesicular disease was experimentally induced in both groups with the duration and magnitude of clinical signs similar between groups. During acute infection [0–14 days post infection (dpi)], SVV was detected by PCR in serum, nasal swabs, rectal swabs, various tissues, and in swabs from ruptured vesicles. From 15 to 30 dpi, virus was less consistently detected in nasal and rectal swabs, and absent from most serum samples. Virus neutralizing antibody was detected by 5 dpi and lasted until the end of the study.

**Conclusion:**

Treatment with an immunosuppressive dose of dexamethasone did not drastically alter the clinical disease course of SVV in experimentally infected nursery aged swine. A greater understanding of SVV pathogenesis and factors that could exacerbate disease can help the swine industry with control and prevention strategies directed against this virus.

## Background

Vesicular disease in swine is recognized by the development of vesicles on the feet, snout, and less frequently in the oral cavity [1, 2]. Nonspecific signs include fever, lethargy, anorexia, and lameness. Known viral causes of swine vesicular disease are vesicular stomatitis virus, swine vesicular disease virus, vesicular exanthema of swine virus, and importantly, foot-and-mouth disease virus (FMDV). FMDV causes one of the most highly contagious diseases of livestock that can result in devastating economic losses to the agricultural industry and disruption of the human food supply [3, 4]. Since vesicular diseases of swine are indistinguishable in the field, each case must be treated as if it was an FMDV infection, which triggers a significant response in countries where FMDV is not endemic. Although rare, swine vesicular diseases have occurred in countries without known vesicular viruses or the known causes have been ruled out, resulting in the diagnosis of porcine idiopathic vesicular disease (PIVD).

*Senecavirus A*, commonly known as Seneca Valley virus (SVV), is a non-enveloped, single-stranded RNA virus in the family *Picornaviridae* first identified as a tissue culture contaminant in 2002 [5]. A retrospective analysis confirmed that since the late 1980s, SVV has been sporadically isolated from swine samples in the United States (US) [6], and detected by PCR in more recent cases of PIVD [7, 8]. Although early attempts to induce vesicular disease with field SVV isolates were unsuccessful [6, 9–11], it was presumed that SVV was the causative agent.

In late 2014, outbreaks of vesicular disease in finishing swine, and in sow farms with concurrent reports of increased neonatal mortality occurred in multiple states in Brazil [12, 13]. As part of the diagnostic investigations ruling out known causes of vesicular disease in swine, SVV was identified by PCR and virus isolation in multiple samples from affected animals. In July 2015, similar outbreaks began in the US and SVV was also detected in those cases [14–17]. Collectively, the association of SVV with vesicular disease in Brazil and the US provided strong support for SVV as the causal agent. This was confirmed with the fulfillment of Koch’s postulates in 9-week old pigs using a 2015 SVV isolate from the US [18]. Since that report, vesicular disease was also experimentally reproduced with SVV infection in nursery pigs [19] as well as in finishing-aged swine [20]. Although SVV was rarely detected in North America prior to the 2014/2015 unprecedented emergence of PIVD in Brazil and the United States, it has been detected many times since then in the respective countries as well as recent novel case reports in Canada [21], China [22–24], Thailand [25], and Colombia [26]. Interestingly, viruses from these recent outbreaks are genetically similar sharing > 94% nucleotide identity at the full-length genomic level.

In an early PIVD report there was speculation that stressful events in the field may predispose pigs to SVV clinical disease; e.g., after transportation to slaughter [8]. Similar observations in the 2014/2015 SVV cases supported this assumption which led to the original experiment using an immunosuppressive model to test the hypothesis that administration of a synthetic glucocorticoid would exacerbate the SVV infection in swine. Surprisingly, both non-dexamethasone treated pigs as well as dexamethasone treated pigs developed vesicular disease of comparable severity. The acute phase of the vesicular disease in the non-dexamethasone SVV-challenged pigs was previously reported [18]. This manuscript describes the kinetics of the SVV infection and the comparison between the dexamethasone and non-dexamethasone treated pigs.

## Results

### Clinical and microscopic observations

All pigs were free from signs of vesicular disease prior to challenge, and all control pigs appeared normal throughout the experiment. One pig in the Dex-SVV group became anorexic at 2 dpi and was removed from the experiment because it was not competitive in a group environment. The pig’s health continued to deteriorate and it died 2 days post removal from the group. Although no definitive cause of death was determined, it is believed SVV did not contribute to the illness and death since the only clinical signs recognized in the other pigs was transient lameness.

A mild transient lameness was recognized in 2–3 pigs from both the Dex-SVV and SVV groups on 2 and 3 dpi. No gross abnormalities in behavior or appearance were observed in pigs euthanized on 2, 4, 8, and 12 dpi for necropsy.

**Fig. 1:**
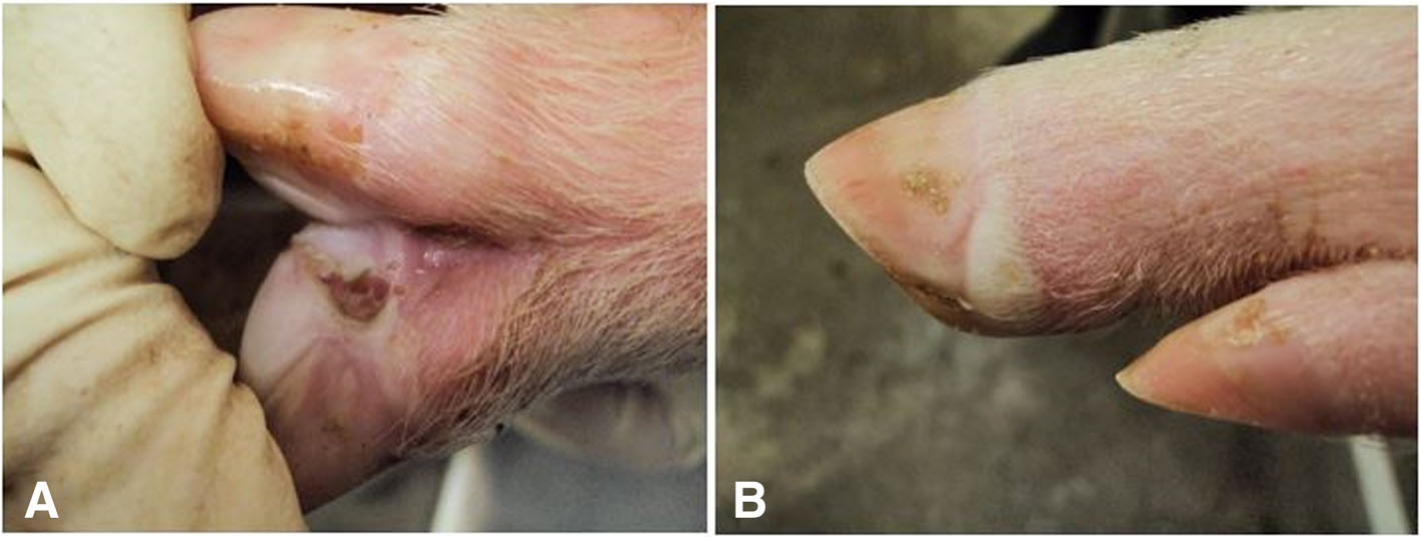
Vesicular lesions from 9-week-old swine. **a**) Ruptured vesicle in the interdigital space. **b**) Intact vesicle on the lateral coronary band

The acute lesions for the SVV pigs were previously described [18]. The lesions that developed in the Dex-SVV group were indistinguishable from the SVV group and are briefly described below. At the 4 dpi daily observation, cutaneous lesions were detected in 8/11 Dex-SVV pigs (72.7%) and 7/16 SVA pigs (43.8%). Cutaneous lesions consisted of small vesicles (about 3 mm × 3 mm) and/or erosions first noticed at 4 dpi in the interdigital spaces and coronary bands of one or more feet. At 5 dpi, all Dex-SVV pigs were observed with vesicular lesions and 14/15 SVV pigs had at least one lesion. Lesions were recognized as small, pale or blanched areas of swelling on the coronary band that would grow in size, thicken and become raised (Fig. 1). Usually, the skin would wear away leaving an erosion or ulcer that could coalesce with adjacent lesions. Snout lesions, when present, were mostly recognized as an elliptical erosion (3 mm × 5 mm) that was on the dorsal ridge of the snout which quickly healed. No new coronary band lesions were recognized after 6 dpi at which time the lesions began to heal.

**Fig. 2:**
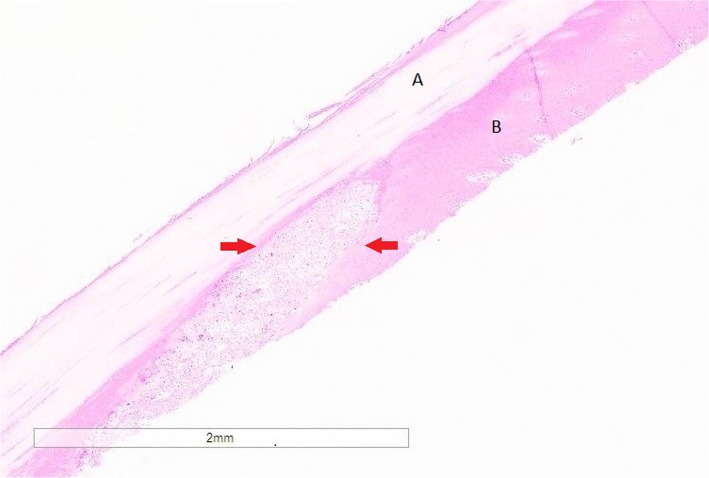
Microscopic lesions from the coronary band. Cut section of the epidermis with the (**a**) stratum corneum and (**b**) stratum spinosum. Red arrows point to an oval shaped vesicle disrupting the stratum spinosum, which contains small numbers of neutrophils, lymphocytes, varying amounts of fibrin, edema admixed with necrotic and cellular debris

Microscopic examination of coronary band tissue sections from the pigs euthanized revealed lesions in the 4 and 6 dpi pigs. Extensive areas of epidermis, predominantly stratum spinosum, was affected and effaced by multifocal to coalescing vesicles, containing fibrin, edema, necrotic debris and infiltration of lymphocytes and plasma cells (Fig. 2). Occasionally, these vesicles progressed to pustules, which were characterized by degenerate neutrophils admixed with variable amounts of fibrin, cellular and karyorrhectic debris. A few of these inflammatory cells were multifocally observed in the superficial dermis (beneath the affected epidermis).

### SVV RNA detection

**Fig. 3:**
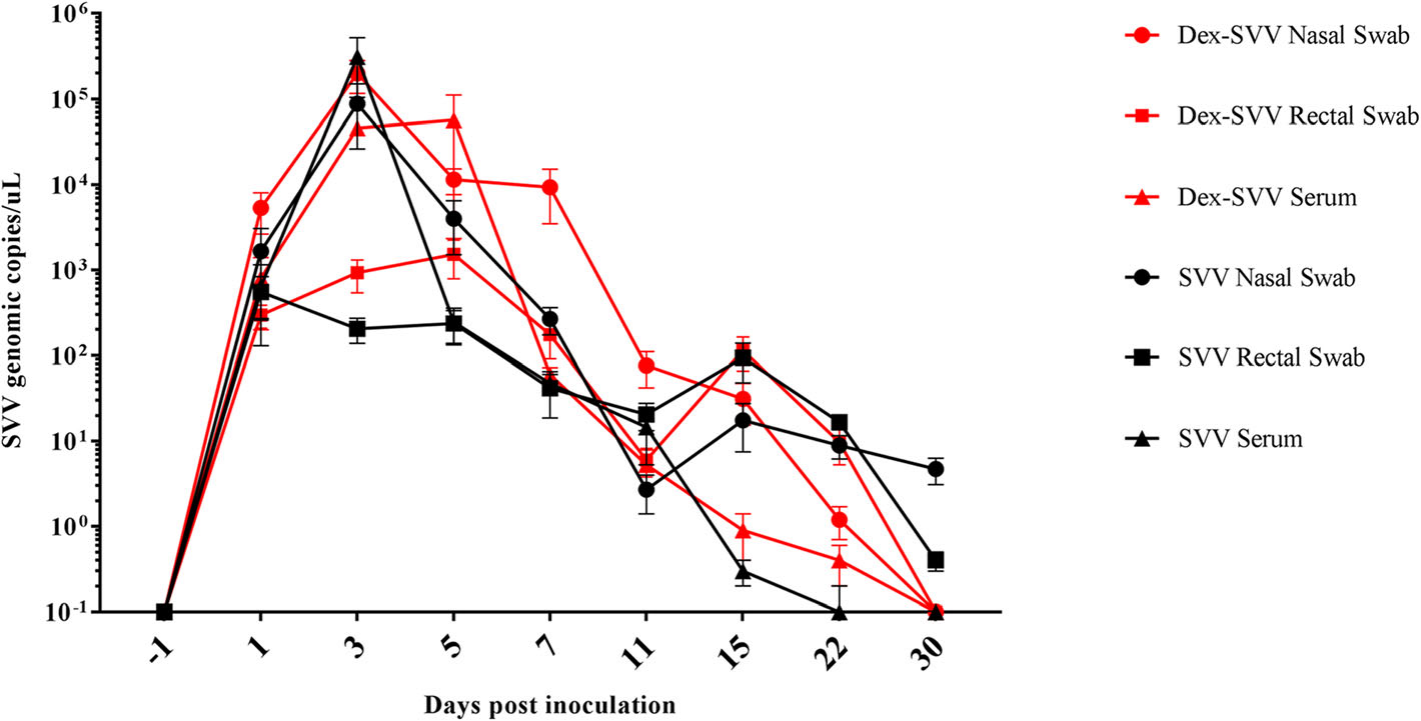
SVV infection dynamics. Virus shedding in nasal and rectal swabs and viremia levels detected by RT-qPCR. Quantity of viral RNA is expressed as genomic copies per microliter. Error bars represent the standard error of the mean. Red data lines represent the Dex-SVV group and black data lines represent the SVV group. Nasal swabs are designated with a circle, rectal swabs with a square, and serum with triangles. No viral shedding or viremia was detected in control animals

Selected samples from control animals all tested negative for SVV RNA by PCR. In serum samples of both Dex-SVV (*n* = 11) and SVV (*n* = 12) groups SVV RNA was detected in pigs as early as 1 dpi (Fig. 3). For the Dex-SVV group, peak viremia occurred at 5 dpi with a mean value of 5.7 × 10^4^ genomic copies per microliter (GC/μL) of serum. Peak of viremia was observed at 3 dpi in the SVV group with a mean value of 3.1 × 10^5^ GC/μL of serum. Though different peaks were observed, no statistically significant differences were detected in the magnitude of viremia between Dex-SVV and SVV groups. Mean SVV RNA concentration in serum in both groups decreased over time with minimal amounts detected at 15 (0.6 GC/μL) and 22 dpi (0.3 GC/μL).

In nasal swabs, SVV RNA was detected in one or more pigs at all time-points of the study, with a peak mean value of 2.0 × 10^5^ GC/μL in the Dex-SVV group and 8.8 × 10^4^ GC/μL in the SVV group at 3 dpi (Fig. 3). By 30 dpi, GCs were found at low levels in all animals of the SVV group and only three animals were PCR positive in the Dex-SVV group. There was no statistically significant difference in GC/uL levels between the Dex-SVV and SVV group.

In rectal swabs, SVV RNA levels were approximately 2 to 3 logs lower than those in nasal swabs (Fig. 3). An observable peak occurred at 3 dpi for the Dex-SVV group (9.5 × 10^2^ GC/μL) and at 5 dpi for the SVV group (2.6 × 10^2^ GC/μL) with statistically significant differences between the two groups detected at 3 and 5 dpi (*p* < 0.05). In addition, both experimental groups had a transient increase in SVV RNA levels from 11 dpi to 15 dpi.

**Fig. 4:**
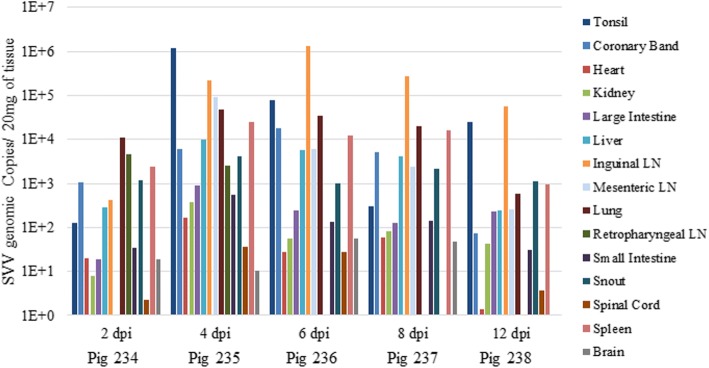
Tissue distribution of SVV. Viral load present in selected tissues from necropsied pigs on 2, 4, 6, 8, and 12 dpi respectively. Quantity of viral RNA was determined by RT-qPCR and expressed as genomic copies per 20 mg of tissue

SVV RNA was detected in almost every tissue tested in each pig necropsied at 2, 4, 6, 8, and 12 dpi (Fig. 4). In general, the lowest RNA levels were detected in pig 234 (2 dpi) with peak RNA levels detected in pig 235 (4pi) followed by a reduction over time for pigs 236 (6 dpi), 237 (8 dpi), and 238 (12 dpi), respectively. SVV RNA was found at much higher concentrations in the tonsil (7.83 × 10^5^ GC/μL at 4 dpi) and inguinal lymph nodes (6.55 × 10^5^ GC/μL at 6 dpi) compared to the other tissues.

### Serological responses

**Fig. 5:**
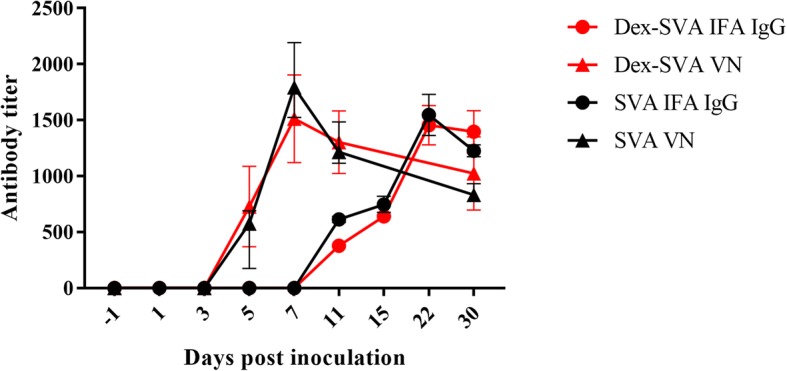
Antibody response to SVV infection. Indirect immunofluorescence assay measured IgG antibody response to SVV infection. Virus neutralization assay measured neutralizing antibody response to SVV. Red data lines represent the Dex-SVV group and black data lines represent the SVV group. IFA titers are depicted with circles while VN titers are designated with triangles. Error bars represent the standard error of the mean

All 0 dpi sera samples were negative for antibodies against SVV by IFA and VN assays. All challenged pigs developed IgG IFA antibodies against SVV by 11 dpi with titers ranging from 1:320 to 1:640. Pigs from both groups had a similar IgG response throughout the study with most having a titer of 1:1280 on 30 dpi (Fig. 5). All but 2 pigs in the Dex-SVV group developed measurable neutralizing antibodies by 5 dpi. VN titers peaked in both groups at 7 dpi. Again, the neutralizing antibody response was similar for both the Dex-SVV and SVV groups over the course of the experiment as shown in Fig. 5. Control pig sera had VN titers ≤1:4.

## Discussion

Our hypothesis for this study was that stress induced through an immunosuppressive dose of dexamethasone would exacerbate clinical disease in SVV challenged pigs compared to those not treated with dexamethasone. This experimental question was derived from failed attempts prior to 2016 to reproduce vesicular disease with SVV, and the observations that PIVD cases were associated with times of stress such as transportation of finishing pigs to market [8] and congregations of show pigs. In this study, SVV infection induced prolific vesicular lesions in both the SVV and Dex-SVV treatment groups. The onset, character, and duration of the lesions were similar between groups indicating the dexamethasone treatment did not dramatically alter the clinical disease of pigs after SVV challenge. More pigs developed vesicular lesions earlier in the Dex-SVV group, but by 5 dpi all but one pig in this experiment developed clinical signs. For the purposes of this discussion, both groups will be discussed as one unless noted otherwise.

Development of vesicular disease in both challenge groups was surprising since experimental infections reported before 2016 were unable to induce clinical disease [6, 9–11]. Vesicular disease reproduced in this study was compatible with early field reports of SVV-related PIVD in finishing-aged pigs and sows [12, 13, 17], and similar to recent reports of experimentally induced vesicular lesions with US wild-type virus in 3-week-old pigs [19] and 55 kg pigs [20]. Collectively, this research may provide insight into the kinetics of SVV-associated vesicular disease that can provide evidence for recommendations for diagnostic sampling and possible control strategies.

In this study, lesions were first noted in the interdigital spaces when pigs were observed at 4 dpi. It is likely the onset of foot lesions began at an earlier time-point, given that by 4 dpi some pigs already had both intact and ruptured vesicles on their feet. The incidence of foot lesions peaked about 5–6 dpi and most lesions had resolved in about a week after onset of clinical signs. Our timeline of vesicular lesion development is similar to that reported by Joshi et al. in finishing pigs experimentally inoculated with SVV where lesions were first observed at 4 dpi [20]. In contrast to onset and incidence of coronary band lesions, snout lesions observed in this study were first recognized around 7 dpi in just a few pigs as one or two small elliptical plaques on the dorsal ridge of the snout. In comparison, a higher incidence of snout lesions was reported in older [20] and nursery age pigs [19].

Viremia and viral shedding were measured by RT-PCR to gain a better understanding of SVV infection kinetics. PCR results revealed a rapid onset of viremia with many pigs testing positive for SVV in serum by 1 dpi. Peak RNA concentration in sera occurred at 3–5 dpi followed by a rapid decrease becoming almost undetectable by 15 dpi. Viral shedding also had a rapid onset with both nasal and fecal swabs testing positive for SVV at 1 dpi. The Dex-SVV group had statistically higher levels of SVV detected by PCR at 3 and 5 dpi in the rectal swabs. Virus was detected longer in nasal swabs than fecal swabs, especially in the SVV group. By 30 dpi most pigs in either group were SVV-negative on rectal swab, but some of the SVV group were still nasal swab positive for viral RNA, though there was no statistical difference between groups. A similar shedding pattern was observed by Joshi et al. who reported that oral, nasal, and fecal swabs became PCR negative by 28 dpi in finishing pigs [20]. Oral swabs were not collected in the present study, but others have reported a greater amount of virus detected and longer shedding compared to fecal or nasal swabs [19, 20].

In the pigs euthanized from 2 to 12 dpi, 4 dpi was the time point with highest detection of SVV nucleic acids in tissues. In general, the tissues with the greatest SVV RNA presence were coronary band epithelia, tonsils, inguinal and mesenteric lymph nodes, lung, liver, and spleen. In addition, most tissues had higher SVV RNA concentrations than serum, which suggests that viral concentrations in the tissues is not solely due to blood contamination. A similar tissue distribution was reported in mid-finishing swine during acute infection in addition to lymph nodes, spleen, intestine, kidneys, and tonsils testing positive by PCR 38 days after challenge [20]. Similarly, diagnostic investigations conducted in sow herds affected by vesicular disease and neonatal mortality in the US and in Brazil described detection of SVV nucleic acids in most tissues of piglets including the small intestine, tonsil, lung, heart, liver, spleen, kidney, myocardium and cerebellum [27, 28]. Previous field case reports have documented detection of SVV in scrapings of ruptured vesicles and ulcerative lesions by PCR in sows and finishing pigs [7, 12, 13, 28]. Based on PCR results in our study, vesicular fluid/swabs had the highest concentration of virus compared to other samples.

The onset of the humoral immune response was similar for both groups with antibody titers first being detected at 5 dpi by VN assay and 11 dpi by IFA assay. The onset and peak of antibody titers was more rapid for neutralizing antibody response compared to IgG measured by IFA assay. Similarly, Joshi et al. detected VN antibody as early as 5 dpi, which was also described by Yang et al., and SVV-specific IgG antibodies by 10 dpi [11, 20]. Chen et al. reported neutralizing antibody response as early as 3 dpi in weaned pigs [19]. Aforementioned studies have credited the early neutralizing antibody response for the quick decline in viremia reported in various aged swine. The duration of a SVV humoral immune response is unknown though unpublished work has demonstrated sterilizing immunity in gilts challenged five months after initial exposure to wild type SVV (Buckley, unpublished observations).

Seneca Valley virus is in the same virus family as FMDV and shares many structural characteristics as well as a similar clinical presentation. Vesicular lesions can develop within 24–48 h of FMDV challenge [2, 3, 29], and are most commonly found on the coronary bands [30]. FMDV can also be detected in serum as soon as 24 h after exposure followed by gradual decline over a couple of weeks [31]. Antibodies against FMDV have been detected by ELISA around 7 dpi (IgM) and 14 dpi (IgG, IgA) in swine either inoculated or in direct contact [32]. Although vesicular lesions contain the highest concentration of FMDV, virus replication does occur in other tissues, e.g., in the tonsil [31]. This study and others have consistently found high concentrations of SVV nucleic acids present in the tonsil not only acutely, but also in convalescent swine [15, 20], which may provide evidence for the tonsil being a primary site of viral replication. The kinetics of the SVV infection are similar to FMDV; however, it is unknown if SVV is as infectious as FMDV. Replication and shedding of SVV from the tonsil into the oropharyngeal cavity probably plays an important role in producing SVV-positive oral secretions, and may contribute to contamination of the nasal passages and the finding of SVV-positive nasal swabs. However, the possible replication of SVV in nasal mucosa has not been ruled out.

Unpublished observations from our laboratory support recent field reports describing environmental contamination with SVV nucleic acid [33], which combined with normal pig behavior such as rooting and inquisitive chewing, could cause false-positives in oral fluid samples. The extended presence of SVV in tonsil tissue may help explain the apparent longer shedding patterns reported for oral fluids and nasal swabs when compared to fecal swabs [19, 20]. Further study will be required under experimental conditions to characterize the duration of shedding of infectious virus from the oral cavity.

Results from Chen et al., Joshi et al., and the present study can certainly be used for diagnostic recommendations for nursery to mid-finishing pigs that will be presented dogmatically for simplicity. Viral RNA can be detected in serum from at least 2–7 days, in feces (rectal swabs) from at least 2–15 days, in nasal swabs from at least 2–15 days, and in oral fluids from at least 2–21 days post infection. During the acute infection viral RNA can be detected in many tissues for at least 2–12 days post infection and can also serve as potential diagnostic samples. Finally, swabs of vesicular lesions may serve as the best sample to collect during clinical disease to reliably detect the presence of SVV due to the large quantities of virus found in that location. Lastly, it is expected that sows infected with SVV will have similar infection kinetics to nursery and mid-finishing swine and sample recommendation listed above would also pertain to mature animals.

Compared to other species, swine are relatively resistant to glucocorticoids [34, 35]; however, their use has induced recrudescence of latent pseudorabies virus in swine indicating glucocorticoids can alter or suppress a pig’s immune system [36]. Dexamethasone administration in this study was based on previous studies that successfully induced recrudescence of wild-type and attenuated PRV vaccine [36], and presumably this dosing schedule would be a substitute for stress that would alter the immune system allowing for enhanced vesicular disease. The Dex-SVV group had more pigs observed with vesicular lesions on 4 dpi, but by 5 dpi all except 1 pig was effected in both groups. In addition, there were higher concentrations of SVV detected in rectal swabs on 3 and 5 dpi compared to the SVV group. It is unknown if a higher glucocorticoid dose might have altered the disease course more significantly, but such efforts may not be needed with contemporary SVV isolates based on this experiment and the previous successful reports [19, 20].

The relative ease in which vesicular disease was produced in both groups of pigs in this study, and in the experiments of Chen et al. and Joshi, et al. provides a basis for a variety of questions and assumptions about contemporary SVV isolates, and why attempts to reproduce vesicular disease prior to 2016 were unsuccessful. First, the genetic similarity of current SVV isolates from Brazil, U.S., Canada, China, Thailand, and Colombia suggests a recent common ancestor which might explain the timing of newly recognized outbreaks across separate continents. Second, there may be a mutation in SVV that increased the likelihood that contemporary viruses are more likely to induce vesicular disease than viruses isolated before 2014. If either of these factors played a role in the emergence of the mini SVV epidemics, then there must be some explanation for the relatively recent distribution of the common virus among continents. However, an increased awareness of potential SVV-related vesicular disease may be contributing to the current observations. For example, the increase in reports of clinical disease associated with SVV isolates in China [24] and Thailand [25] that are more distantly related genetically to the contemporary Brazilian and US isolates suggests current clinical cases are not completely dependent on the emergence of a new lineage of virus. Third, there may be a novel cofactor(s) such as another infectious agent allowing disease to develop, which if true, must also have been transmitted to different continents. Fourth, there may be a genetic predisposition to clinical disease that is more prevalent now when compared to pre-2014 swine, and this trait has been slowly building in swine herds in countries reporting recent events. In addition, there may be an age-dependent effect on clinical presentation of disease which makes it more difficult to observe disease in young pigs compared to mature swine. The focal nature of vesicular disease demonstrates a discrete viral tropism for a cell type in a specific anatomical site, i.e., coronary band tissue. Such a condition might involve an increased density of this cell type in older swine, or this tissue may be more prevalent in one genetic line compared to another which would contribute to the presence of gross lesions. Lastly, there is the possibility that two or more of the above factors contributed to the emergence of the mini SVV epidemics.

## Conclusions

This study adds to the growing body of knowledge about the pathogenesis of SVV in swine that has focused on understanding the acute infection (clinical disease, viremia, viral shedding, virus distribution in tissues, and immune response). This work has demonstrated the need to investigate the duration of immunity in convalescent swine and the spectrum of immunity against historical and contemporary strains to discern why an apparent change has occurred in the ecology of SVV in swine. The clinical description and kinetics from this study can also help diagnosticians and veterinarians improve strategies to control this disease and differentiate it from other vesicular diseases in swine.

## Methods

### Cells and virus

A swine testicular (ST) cell line (ATCC® CRL-1746; American Type Culture Collection, Manassas, VA) was cultured at 37 °C and 5% CO_2_ in minimum essential medium (MEM, MilliporeSigma, St. Louis, MO) supplemented with 10% fetal bovine serum (FBS, AtlantaBio, Flowery Way, GA), 1x gentamicin, and 1x glutamax (Life Technologies, Carlsbad, CA). NCI-H1299 cells (ATCC® CRL-5803) were cultured under the same conditions in Dulbecco’s modified Eagle’s medium (DMEM, MilliporeSigma) supplemented with 10% FBS (AtlantaBio).

SVA15-41901SD was isolated on ST cells from samples collected from a barn of finishing pigs in South Dakota that developed vesicular lesions during the summer of 2015 [15]. The second and third passage of the virus were combined to create a larger volume of working stock virus which was utilized for inoculum and laboratory assays. The stock virus had a titer of 4.75 × 10^7^ plaque-forming units (PFU)/ml and was tested for purity by next-generation-sequencing that only detected SVA.

### Animals and experimental design

Forty-nine conventionally-raised weaned pigs were purchased, randomly assigned ear tags and housed at the USDA-ARS-NADC campus in accordance with Institutional Animal Care and Use Committee guidelines (ACUP #2867) until 9 weeks of age at which time 29 pigs received SVV at a dose of 4.75 × 10^7^ PFU/animal via intranasal inoculation. A LMA® MAD Nasal™ Intranasal Mucosal Atomization Device (Teleflex; Morrisville, NC) was used for delivery of about 2.5 mL of the atomized inoculum into each nostril for a total of 5 mL. Twelve pigs (Dex-SVV group) based on sequential ear tag numbers were given an immunosuppressive treatment of dexamethasone (AgriLabs; St. Joseph, MO) intramuscularly in the neck for 5 days prior to challenge as follows: day 1, 2.3 mg/kg of body weight twice a day (BID); days 2–5, 1.1 mg/kg BID. Seventeen pigs (SVV group) did not receive any treatment before challenge. The remaining 20 pigs were unchallenged control pigs (Control group). Each group was housed in a separate animal-biosafety-level 2 room, with at least 17 square feet/pig and constant access to feed and water. One pig each from the SVV group was euthanized on 2, 4, 6, 8, and 12 days post inoculation (dpi). At the time of euthanasia, the animal was physically restrained for the intravenous administration of a barbiturate (Fatal Plus, Vortech Pharmaceuticals, Dearborn, MI) following the manufacturer label dose (1 mL/4.54 kg). At the conclusion of the study the remaining pigs were also euthanized in the same manner.

### Sample collection

Progression of clinical disease for each pig was assessed by daily observations for lameness and vesicular lesions. Antemortem sampling consisted of collection of whole blood in serum separation tubes (BD Vacutainer®, Franklin Lakes, NJ), and nasal and rectal swabs. Swabs (Puritan Medical Products, Guilford, ME) were collected and immersed in 3 ml of serum-free MEM (MilliporeSigma). Samples were collected on 0, 1, 3, 5, 7, 11, 15, 23, and 30 dpi from the SVV and Dex-SVV groups. Pigs from the control group were sampled at 0, 7, 15, and 29 dpi. When vesicles were observed, vesicular fluid collection was attempted via aspiration or swabs. Blood tubes were centrifuged to harvest the serum. Sera, vesicular fluids, and swab tubes were stored at − 80 °C until time of testing.

In the pigs euthanized on 2, 4, 6, 8, and 12 dpi, all postmortem examinations were performed immediately after euthanasia, and tissue specimens collected included snout and coronary band epithelium, tonsil, lymph nodes (retropharyngeal, mesenteric, and/or inguinal), lungs, heart, liver, spleen, kidney, small and large intestine, brain and spinal cord. For each anatomically defined specimen, approximately 10 g of tissue was collected and placed into a self-sealing plastic bag on dry ice for transfer within 2 h to a − 80 °C freezer. Tissues were thawed and 20 mg of each sample resuspended in 5 mL 1x PBS (Thermo Scientific, Waltham, MA) in individual gentleMACS™ M tubes (Miltenyi Biotec, Auburn, CA). Tissue was dissociated using a gentleMACS™ Octo-Dissociator (Miltenyi Biotec, Germany) following the manufacturer’s recommendations. After dissociation, tissue suspensions were aliquoted in 2 mL cryogenic vials (Corning, Corning, NY) and frozen at − 80 °C until time of processing. For histology, skin sections from the coronary band region were collected in 10% neutral buffered formalin. Sections were fixed in formalin for 24 h, routinely processed, paraffin-embedded, sectioned at 4-μm thickness, and stained with hematoxylin and eosin.

### Seneca Valley virus-specific nucleic acid detection

Samples were extracted using the MagMAX™ Pathogen RNA/DNA kit (Life Technologies, Carlsbad, CA) following manufacturer’s recommendations and a MagMAX™ Express instrument 24 (Life Technologies) using program AM1836 (Life Technologies). The viral RNA was eluted in 90 μL of elution buffer. Following extraction, 5 μL of the nucleic acid templates were added to 20 μL of the Path-ID™ Multiplex One-Step RT-PCR reaction master mix (Applied Biosystems, Foster City, CA) for fecal swabs or 20 μL AgPath-ID™ One step RT-PCR kit (Applied Biosystems) for nasal swabs, vesicle fluids, and sera. The primers and probe were designed to target the conserved region between the 5′ untranslated region (5’UTR) and protein L containing nucleotides 602–710 of the SVA genome. The forward primer sequence was 5’-TGCCTTGGATACTGCCTGATAG-3′, the reverse primer sequence was 5’-GGTGCCAGAGGCTGTATCG-3′ and the probe sequence was 5’-CGACGGCCTAGTCG GTCGGT T-3′. The probe was labeled using 6-FAM™ at the 5′ end, ZEN™ internal quencher, and Iowa Black® quencher at the 3′ end (Integrated DNA Technologies, Coralville, IA). Real-time RT-PCR was performed on an ABI 7500 Fast instrument (Life Technologies) run in standard mode with the following conditions: 1 cycle at 48 °C for 10 min, followed by 1 cycle at 95 °C for 10 min, and 40 cycles of 95 °C for 15 s and 60 °C for 45 s. SVA genome RNA copies were calculated based on a standard RNA transcript overlapping the target region.

### Indirect immunofluorescence (IFA) assay

SVV-specific IgG antibody response to SVV challenge was evaluated by an IFA assay. Serum samples were serially diluted 1:2 up to (1:5120). Plates were previously prepared with SVV-infected NCI-H1299 cells overnight and fixed with cold 80% acetone, and stored at − 20 °C. Plates were rehydrated with 200 μL of PBS and 50 μL of diluted serum were added to wells to incubate for 1 h. Plates were washed 3 times and 1:50 diluted anti-swine IgG-FITC antibody (KPL, MD, USA) was added to incubate for 45 min. Again, plates were washed 3 times and wells were observed for fluorescence under a fluorescent microscope. The highest serum dilution with clear and specific staining was considered as the end point (i.e., IFA titer).

### Virus neutralization (VN) assay

Serum samples were heat-inactivated at 56 °C for 30 min and serially diluted 1:4 (up to 1:4096) in MEM in a 96-well plate and repeated in quadruplicate. Each diluted serum was mixed with an equal volume of SVA15-41901SD (200 TCID_50_) and incubated for 1 h at 37 °C. One-hundred microliters of each virus-serum mixture was transferred to respective wells of 96-well plates of ST cells grown to a confluent monolayer and replenished with MEM supplemented with 2% FBS. Plates were read for CPE daily for 4 days. VN titers, based on CPE, were recorded as the highest dilution of serum at which the infectivity of SVA-41901SD was completely neutralized in 50% of the inoculated wells. A back titration of the virus was performed during each test.

### Statistical analysis

Data analyses and graphic representations were performed by using Microsoft Office Excel 2010 and GraphPad Prism 7.03. Statistical analyses of the data were performed using a mixed linear model (SAS 9.4 for Windows XP, SAS Institute Inc., Cary, NC, USA) for repeated measures and a spatial spherical or autoregressive covariance structure was utilized. Linear combinations of the least squares means estimates for GC/uL were used in a priori contrasts after testing for either a significant (*P* < 0.05) effect of treatment or a significant treatment by time interaction. Comparisons were made between treatment groups at each time-point using 5% level of significance (*P* < 0.05) to assess statistical differences.

## Abbreviations

BID: twice a day
CPE: cytopathic effect
DMEM: Dulbecco’s modified Eagle’s medium
dpi: days post infection
FBS: fetal bovine serum
FMDV: foot-and-mouth disease virus
GC: genomic copies
IFA: indirect immunofluorescence
MEM: minimum essential media
PCR: polymerase chain reaction
PFU: plaque-forming units
PIVD: Porcine idiopathic vesicular disease
ST: swine testicular
SVV: Seneca Valley virus
US: United States
VN: virus neutralization
